# Epidemiology and Treatment Outcomes of Renal Cell Carcinoma in Qassim Region, Saudi Arabia: A Retrospective Study

**DOI:** 10.7759/cureus.72748

**Published:** 2024-10-31

**Authors:** Badr Alharbi, Hatim S Alnosayan, Faisal Awadh Al-Harbi, Alwleed M Alaidah, Albaraa Nasser Almoshiqeh, Abdullah Mulfi Alharbi, Emad Alwashmi, Adil Khalaf Altwairgi

**Affiliations:** 1 Surgery, College of Medicine, Qassim University, Buraydah, SAU; 2 Urology, Medical City, Qassim University, Buraydah, SAU; 3 Medicine, Qassim University, Buraydah, SAU; 4 Surgery, Unaizah Medical College, Qassim University, Buraydah, SAU

**Keywords:** ccrcc, comorbidity, kidney tumor, renal cell carcinoma, saudi arabia

## Abstract

Introduction and aim: Renal cell carcinoma (RCC) is an aggressive malignant neoplasm that accounts for 90% of renal cancers with rising incidence worldwide. This study aimed to analyze the demographics and clinical profiles, histopathological presentations, and treatment outcomes of 73 RCC patients at a hospital in Qassim region, Saudi Arabia.

Methodology: This retrospective observational study was conducted at King Fahad Specialist Hospital, Buraidah, from October 2017 to July 2024. Census sampling included all patients diagnosed with RCC. Data on demographics, clinical, and histopathological characteristics were analyzed using SPSS version 26 (Armonk, NY: IBM Corp.).

Results: RCC was found to be more prevalent in males and older patients with common comorbidities such as diabetes and hypertension. Among the histopathological types of RCC, clear cell RCC (ccRCC) is most frequently reported in 60.3% of patients, followed by chromophobe RCC (chRCC) and papillary RCC (pRCC), in 16.4% and 11% of patients, respectively. Fuhrman grade 2 stage was seen in 65.8% of the tumors, indicating its moderately aggressive form. Incidental diagnoses accounted for 45.2% of patients, with 93.2% demonstrating no evidence of metastasis at diagnosis. The primary mode of treatment of RCC was the surgical procedure with laparoscopy, which was undergone in 52.1% of cases.

Conclusion: ccRCC exhibits the highest prevalence among the various forms of renal cancer, with the majority of cases being diagnosed incidentally. Despite the rising incidence, early diagnosis and improved screening strategies are yet to be elucidated to address the growing burden of RCC and its incidental finding rate in the Qassim region.

## Introduction

Renal cell carcinoma (RCC) is one of the most prevalent renal malignancies [1] responsible for approximately 2-3% of all cancers, with 431,000 new diagnoses and around 180,000 deaths reported globally in 2020. It is a silent cancer, with incidence rising every year [1,2]. RCC is the ninth and 14th most common cancer in males and females worldwide, respectively [3]. Most RCCs arise in the renal cortex, consisting of the glomerulus, collecting duct, and tubular apparatus. In contrast, cancers originating in the renal pelvis exhibit similarities to urothelial cancer histologically and behaviorally [4]. Renal cancer screening and diagnosis has become a top research priority internationally due to its significantly large number of asymptomatic patients, high mortality, and increasing incidence [1,5].

Renal cancers have become more prevalent in developed countries during past decades. It is the ninth most common cancer in the United States, with its incidence doubled since 1975 [6]. The majority of RCC patients belong to North America and Europe. In addition, there is also an expected increase in RCC cases in Africa and Asia due to lifestyle changes. The RCC incidence in the United Kingdom increased by 3.1% per year from 1993 to 2014 [7]. RCC incidence ranges from 1/100,000 in females to 2/100,000 in males in India [8]. The prevalence of renal cancer in Saudi Arabia is 4.6% [9].

Despite being the most fatal urological cancer, its risk factors are still yet to be elucidated [10]. Some of the risk factors include gender, age, and race. The average age of RCC diagnosis in the United States is reported to be 55-74 years [4]. In addition, hypertension, diabetes, and obesity are also among the factors contributing to RCC [11]. Most symptomatic older patients experience loin pain, diabetes, and hypertension compared to younger ones [12]. Ethnicity plays a significant role in RCC incidence in the United States, showing a higher incidence in African Americans and Native Americans compared to Asian Americans [13]. This shows that racial differences and other factors such as diet, education, exercise, and medical facilities also contribute to RCC [14]. In addition, minor factors such as chronic kidney disease, end-stage renal disease, acquired renal cystic disease, viral hepatitis, high levels of low-density lipoprotein (LDL), and genetic diseases are also found to be associated with RCC. Other risk factors include prolonged use of palliatives and processed red meat, low physical activity, and exposure to trichloroethylene and cadmium [14]. Some studies have also indicated that overweight RCC patients have a better prognosis, contradicting the role of obesity in RCC [14].

Renal cancer is a heterogeneous disease divided into three sub-types by the World Health Organization (WHO) based on genetic, molecular, and morphological characteristics. These subtypes include clear cell RCC, constituting 70-90%; papillary cell RCC, 10-15%; and chromophobe RCC, 3-5% of renal malignancies. ccRCC and pRCC occur in proximal tubules, while chRCC originates from distal tubules of the kidney. Around 5% of ccRCCs are due to hereditary disorders, while 95% are sporadic [15]. Out of the total RCCs, pRCC, ccRCC, and chRCC originating from tubular tissue account for above 90% [16]. However, ccRCC makes up around 75% of the total RCC, with the worst prognosis compared to the other two [16]. The age at diagnosis of ccRCC and chRCC is the same, but of pRCC is significantly higher [13]. Overall, around the world, 4.6 is the average age-standardized rate (ASR) for RCC incidence, where men and women have an average of 6.1 and 3.1 ASR, respectively [17]. The incidence of RCC reported in North America is at 12.2, in New Zealand and Australia at 10.2, and in Europe at 9.5 ASR. Africa, Asia, South Korea, Israel, and Japan showed RCC incidence at 1.8, 2.8, 6.5, 7.5, and 7.6 [17]. Around 180,000 deaths occurred due to RCC in 2020, 64,000 women and 116,000 men, which accounts for 1.8% of all cancer-related deaths. RCC mortality was highest in Latin America and Eastern Europe, with Latvia at 4.3, Uruguay at 4.4, and Slovakia at 4.7 ASR [17]. According to Global Cancer Observatory (GLOBOCAN) data, RCC is more common in men, with cases 1.5 times higher than in women [17]. This might be because the lifestyle of men has more habits which are majorly contributing to cancers. RCC is more common in women than pRCC [13].

Over 50% of RCCs are diagnosed accidentally following random detection of abnormal masses in kidneys during routine imaging testing. Only 30% of patients with RCC exhibit symptoms, and around 20-30% have already undergone metastasis of cancer to other parts of the body at the time of diagnosis [18]. If renal cancer is diagnosed before metastasizing, the survival rate is 93% for five years. However, after metastasis to lymph nodes or abdominal organs, the survival rate declines to 70% for five years, while metastasis to distant body regions reaches 12% [19]. RCC is characterized by abdominal pain, hematuria, fever, anemia-induced fatigue, weight loss, cough, and bone pain. Other physical signs, such as peripheral lymphadenopathy (LAP), abdominal mass, varicocele, and lower limb edema, may also be important for diagnosing RCC [20].

Renal cancer management depends on the stages of the disease, with robot-assisted or laparoscopic surgery for early-stage disease, while immunotherapy, such as immune checkpoint inhibitors (ICIs), or targeted therapy, such as tyrosine kinase inhibitors (TKIs), is used in monotherapy or in combination for advanced-stage disease [21]. However, treatment options depend upon the available healthcare facilities.

Qassim is recognized for its agricultural resources, situated within an oasis region that facilitates the cultivation of dates, fruits, and vegetables, thereby establishing it as one of the nation's principal agricultural hubs. Notwithstanding its absence of significant oil and gas reserves, Qassim's economy has diversified through the expansion of various industries and infrastructural developments. The region's desert climate, limited water resources, and distinctive dependence on agriculture rather than oil extraction may significantly influence lifestyle and environmental factors that affect public health, including the incidence and outcomes of renal cell carcinoma.

Qassim area contributes to approximately 4.3% of the total cancers in Saudia Arabia [22]. A higher annual percentage of cancer (APC) is reported to be 15.3% for renal cancer in Qassim compared to the whole country [22]. Despite an expanding body of research on renal cancer in Saudi Arabia, little focus has been directed toward regional differences, especially in the Qassim region.

Investigating regional disparities in Saudi Arabia, emphasizing the Qassim region, will highlight the customized approaches to understanding and managing RCC in specific geographic areas. This study seeks to investigate the demographics, clinical presentations, staging, management strategies, and outcomes of RCC in the Qassim region, aiming to generate insights that can guide more precise interventions and enhance patient care.

## Materials and methods

Study design, setting, duration, size

This retrospective observational study was conducted at King Fahad Specialist Hospital (KFSH) in Buraidah, Saudi Arabia. This facility serves as a tertiary referral center, receiving patients from various regional medical centers. The study duration was 12 months. A comprehensive sample of all patients diagnosed with renal cancer in the study setting from October 2017 to July 2024 was included.

Selection criteria

The census sampling technique was employed. The study included all patients, irrespective of gender and age, who were diagnosed with renal cell carcinoma (RCC) confirmed by histopathological analysis and who underwent surgical intervention (including laparoscopic radical nephrectomy, open partial nephrectomy, or open radical nephrectomy) with complete medical records available. Exclusion criteria included patients with incomplete or missing medical records, a lack of histopathological confirmation of RCC, and those who did not receive surgical treatment for RCC. Furthermore, patients presenting with metastatic disease originating from other primary cancers, a history of previous or concurrent malignancies, or insufficient follow-up data were also excluded from the study.

Data collection variables

Data were collected on various aspects of renal cancer patients, including patients' demographic characteristics (age, gender, nationality, BMI), lifestyle factors (smoking), associated medical conditions, clinical presentation, histopathological characteristics including (histopathological type, tumor laterality, tumor site, Fuhrman grade, invasion, tumor necrosis, focality, tumor markers, disease staging and progression, treatment details, and outcomes. 

Data management and analysis plan

Data cleaning was conducted to ensure the accuracy and integrity of the dataset. Initially, data were entered into MS Excel (Redmond, WA: Microsoft Corp.) and then cleansed to eliminate errors and discrepancies, including duplicate records, missing values, and outliers. Following data cleaning, statistical analysis was performed using SPSS software version 26 (Armonk, NY: IBM Corp.). Descriptive statistics were used to summarize the data, with frequencies and percentages employed to express the distribution of categorical variables.

## Results

Among the 73 participants, 61.6% (N=45) were male and 38.4% (N=28) were female. The majority were aged over 60 years (37%, N=27), with the majority being Saudi nationals (91.8%, N=67). In terms of BMI, 43.8% (N=32) were overweight, 31.5% (N=23) had a normal BMI, and 16.4% (N=12) were obese. Hypertension (45.2%, N=33) was the most common comorbidity, followed by diabetes (31.5%, N=23) and thyroid disorders (15.1%, N=11). Tumor laterality was nearly equal between the left (50.7%, N=37) and right (49.3%, N=36) sides. Most participants were non-smokers (90.4%, N=66), and 52.1% (N=38) underwent laparoscopic radical surgery (Table 1).

**Table 1:** Demographic and clinical characteristics of the study participants. LUTS: lower urinary tract symptoms

Characteristics		Frequency (%)
Gender	Male	45 (61.6%)
	Female	28 (38.4%)
Age (years)	<30	01 (1.4%)
	>60	27 (37%)
	30-40	11 (15.1%)
	41-50	17 (23.3%)
	51-60	17 (23.3%)
Nationality	Non-Saudi	6 (8.2%)
	Saudi	67 (91.8%)
BMI	Underweight	1 (1.4%)
	Normal	23 (31.5%)
	Overweight	32 (43.8%)
	Obese	12 (16.4%)
	Extremely obese	5 (6.8%)
Associated disorders	Thyroid disorders	11 (15.0%)
	Hypertension	33 (45.2%)
	Diabetes mellitus	23 (31.5%)
Tumour site laterality	Left	37 (50.7%)
	Right	36 (49.3%)
Main presenting complain	Incidental	33 (45.2%)
	Abdominal pain on and off	1 (1.4%)
	Flank mass	7 (9.6%)
	Flank pain	9 (12.3%)
	Flank pain, flank mass	2 (2.73%)
	Flank pain, flank mass, hematuria	1 (1.4%)
	Flank pain, flank mass, leg pain	1 (1.4%)
	Flank pain, hematuria	14 (19.2%
	Flank pain, hematuria, dysuria	1 (1.4%)
	Flank pain, weight loss	1 (1.4%)
	Hematuria	1 (1.4%)
	LUTS	1 (1.4%)
	Weight loss, cough with scant sputum of 3 months duration	1 (1.4%)
Smoker	No	66 (90.4%)
	Yes	7 (9.6%)
The type of surgery was undergone	Laparoscopic radical	38 (52.1%)
	Open partial	03 (4.1%)
	Open radical	32 (43.8%)

The most common histopathological type observed was clear cell renal cell carcinoma, occurring in 60.3% (N=44) cases. This was followed by chromophobe renal cell carcinoma in 16.4% (N=12) cases and papillary renal cell carcinoma in 11% (N=8) cases. Less frequent types included clear cell papillary renal cell carcinoma, found in 2.7% (N=2) of cases, and one case each of eosinophilic renal cell carcinoma, microphthalmia transcription factor (MiT) family translocation renal cell carcinoma, oncocytoma, and pure squamous cell carcinoma (Table 2). Regarding Fuhrman's grading, most tumors were classified as grade 2 (65.8%, N=48), followed by grade 3 (15.1%, N=11), and grade 1 (4.1%, N=3). Grade 4 was seen in 2.7% (N=2) cases and 2.7% of cases (N=2) had no grading information. Additionally, grading was not mentioned in 9.6% (N=7) cases (Table 2).

**Table 2:** Frequency of renal tumor histopathological types. MiT: microphthalmia transcription factor

Histopathological type	Frequency (%)
Chromophobe	12 (16.4%)
Clear cell	44 (60.3%)
Clear cell papillary renal cell carcinoma	2 (2.7%)
Eosinophilic renal cell carcinoma, unspecified	1 (1.4%)
MiT family translocation renal cell carcinoma and papillary renal cell carcinoma	1 (1.4%)
Oncocytoma	1 (1.4%)
Papillary	8 (11.0%)
Pure squamous cell carcinoma	1 (1.4%)
Fuhrman grade	
1	3 (4.1%)
2	48 (65.8%)
3	11 (15.1%)
4	2 (2.7%)
Not found	2 (2.7%)
Not mentioned	7 (9.6%)

**Figure 1:**
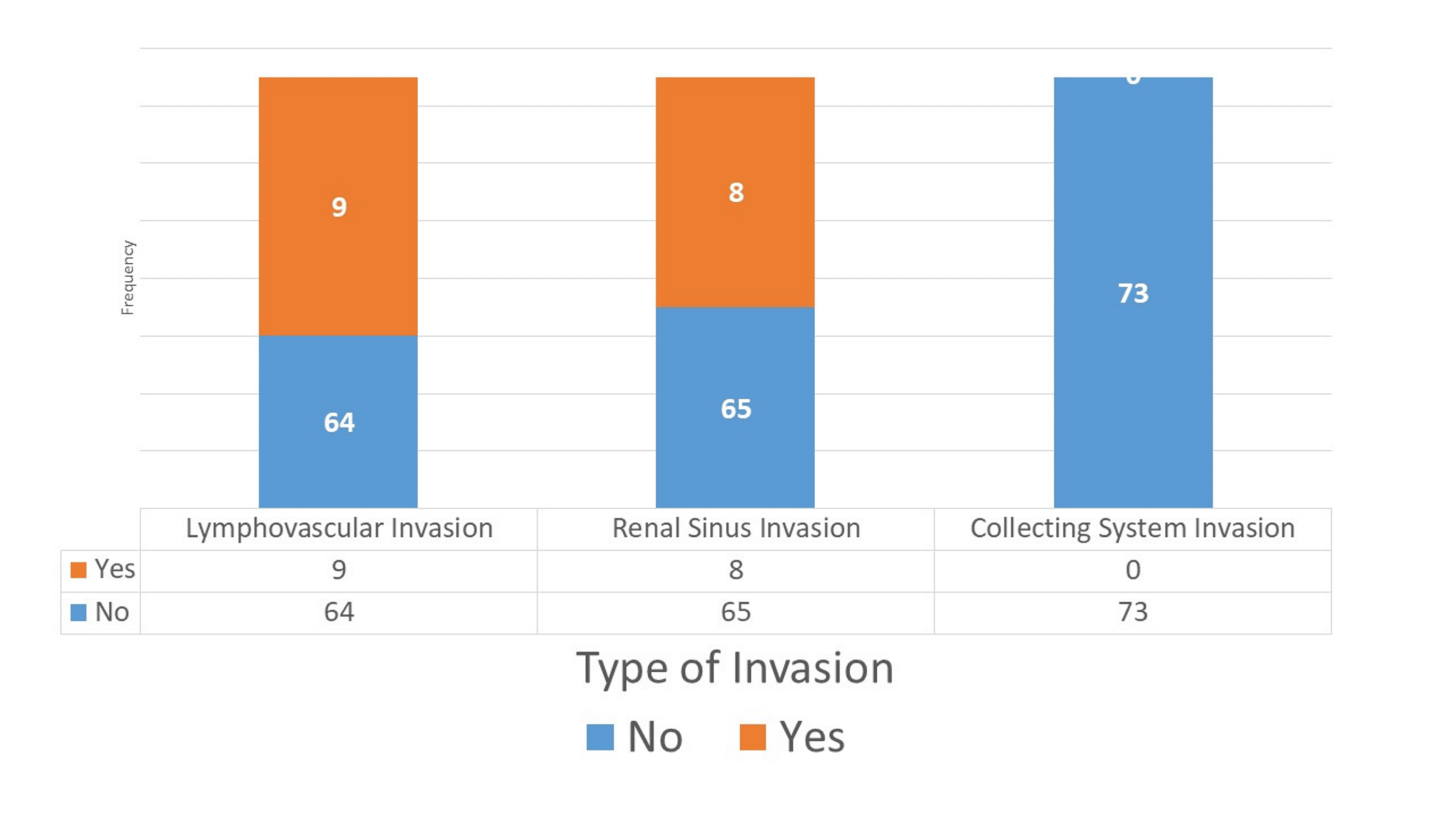
Frequency of different types of invasions.

Out of 73 patients, nine showed lymphovascular invasion, and eight showed renal sinus invasion. No case of collecting system invasion was reported (Figure 1). Metastasis was absent in the majority of cases (93.2%, N=68). Single metastasis was observed in 4.1% (N=3) cases, while multiple metastases occurred in 2.7 (N=2). Tumor necrosis was seen in 45.2% (N=33), whereas it was not detected in 54.8% (N=40) cases. Recurrence was rare, with only 1.4% (N=1) showing recurrence, while the remaining 98.6% (N=72) cases showed no recurrence (Table 3).

**Table 3:** Frequency of metastasis, tumor necrosis, and recurrence.

Metastasis	Frequency
None	68 (93.2%)
Single	03 (4.1%)
Multiple	2 (2.7%)
Tumor necrosis	
Not seen	40 (54.8%)
Seen	33 (45.2%)
Recurrence	
No	72 (98%)
Yes	1 (1.4%)

**Figure 2:**
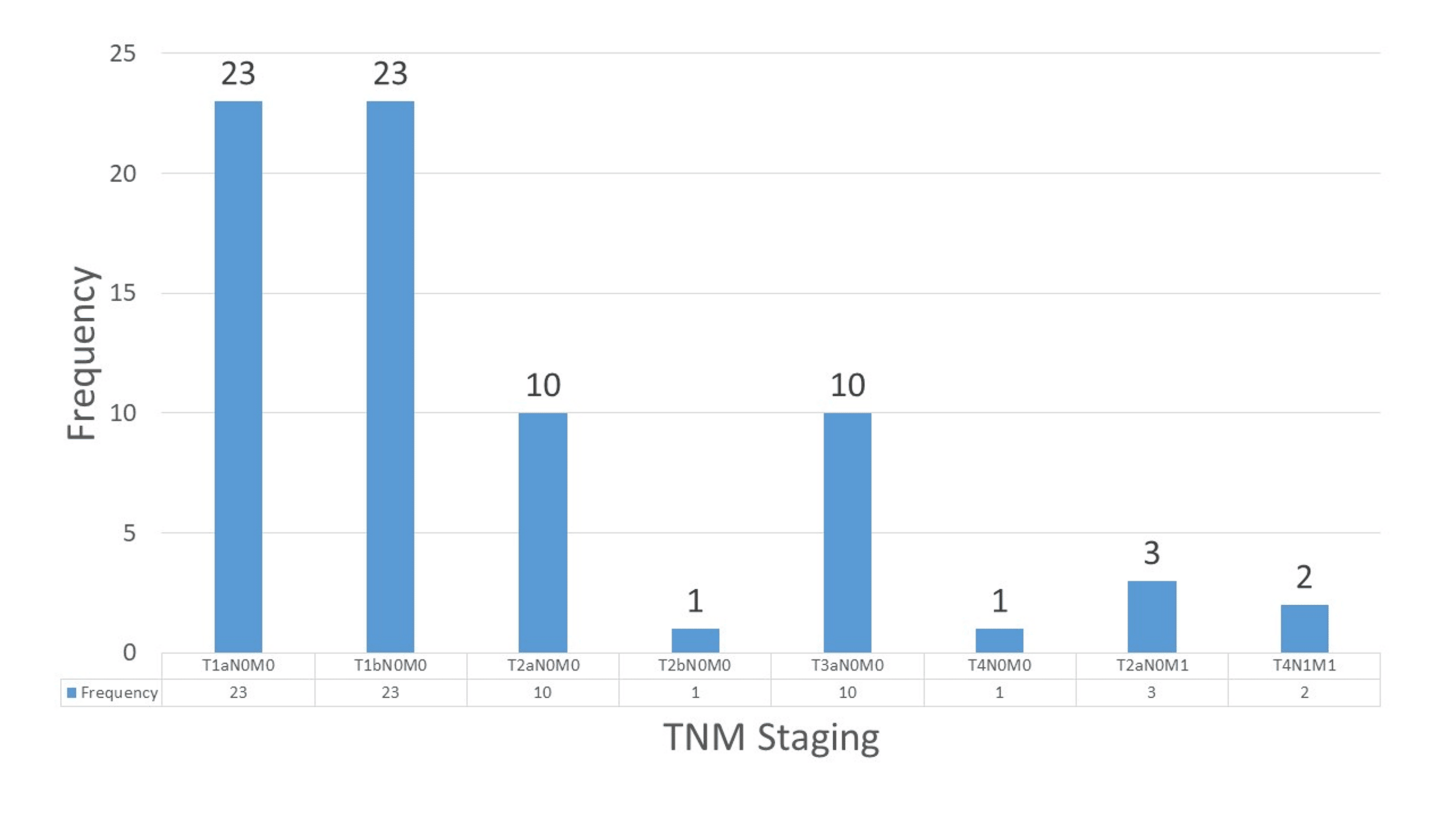
Distribution of TNM stages. TNM: tumor, node, metastasis

The most common stages were T1aN0M0 and T1bN0M0, each occurring in 31.5% (N=23) cases. T2aN0M0 was observed in 13.7% (N=10) cases, while T3aN0M0 was also seen in 13.7% (N=10) cases. Less frequent stages included T2bN0M0 and T4N0M0, each with one case (1.4%). Metastatic stages were rare, with T2aN0M1 observed in 4.1% (N=3) cases and T4N1M1 found in 2.7% (N=2) (Figure 2).

## Discussion

Renal cell carcinoma is an aggressive but silent cancer that accounts for 90% of renal cancers and is rising in prevalence every year. This study demonstrates that the prevalence of renal cancer is higher in males than females, older individuals, and patients with other comorbidities such as diabetes, obesity, and hypertension. Thirty-eight percent of cancer patients have been reported to have hypertension as the most commonly occurring comorbidity [23]. The increasing prevalence of RCC and hypertension might be because of the following two reasons: the occurrence of hypertension as a risk factor of RCC, and RCC can also be caused by nephrectomy, paraneoplastic syndrome, and targeted therapies [24-26]. Although no association was found between diabetes and RCC in the Vitamins and Lifestyle (VITAL) study, an increased risk of RCC was observed in diabetic women and men with high glucose in the Nurses' Health Study [2,27].

In the present study, it was found that 61.6% of the individuals diagnosed with renal cell carcinoma (RCC) were male, consistent with prevailing global patterns. Furthermore, a separate investigation noted a consistently higher incidence of RCC in males, ranging from 1.5 to 2 times higher, across diverse age groups and countries [28]. This may be attributed to lifestyle factors such as smoking in men. However, only 9.6% of participants in the cohort were reported to have a smoking habit, in contrast to the global average. Interestingly, in the population of the Netherlands, a weak correlation was noted between a healthy lifestyle and RCC occurrence [29].

Various factors likely impact regional variation in RCC prevalence, progression, recurrence, and elimination. A constant rise in the global incidence of RCC is observed, mainly in developed countries such as the United States and Europe, but a very slightly increasing slope is observed in the cases in Asia, Africa, and the Middle East owing to increasing comorbidities and rapid lifestyle changes. Overall, 31.5% of patients had diabetes, and 45.2% had hypertension as a risk factor for RCC. The high prevalence of the disease in obese and overweight participants, 16.4% and 43.8%, respectively, confirms the correlation between RCC and obesity. Despite the known association of obesity with RCC, a better prognosis is reported in obese patients compared to individuals with normal weight, creating an obesity paradox. However, an association of obesity with an enhanced risk of fatal RCC in non-metastatic disease has also been reported. In addition, clinical outcome is also predicted by weight loss from pre-diagnosis to post-diagnosis [30]. One explanation of this obesity paradox might be the early detection of low-grade renal incidentalomas in obese patients while diagnosing other diseases. Higher BMI is also linked with low Fuhrman grade and tumor stage [31].

Diet and other lifestyle factors have shown a significant contribution to RCC. Although no significant link between dietary fibers and RCC has been reported, yet increased intake of vegetables and vitamin C, moderate consumption of alcohol in Europeans, and cruciferous vegetables in North Americans have a protective role against RCC [32]. Development of RCC has been reported in less than 5% of the patients, primarily females, who had tuberous sclerosis complex [33].

Similar to previous studies, the highest number of patients were seen having clear cell RCC (ccRCC), accounting for almost 60.3% of the total cases. A total of 11% of participants had papillary RCC, and 16.4% had chromophobic RCC, which mirrors the already published studies [34]. Second-grade Fuhrman tumor was observed in 65.8% of cases, which indicates a disease of moderate aggressive level, suggesting the presence of a notable incidence of intermediate-level RCC in the Qassim region with a substantial progression. Overall, 15.1% of patients were diagnosed with third-grade Fuhrman tumors with severely aggressive disease, while 2.7% were in fourth grade with poor recovery outcomes. Only 4.1% of participants had a first-grade tumor, which has a good prognosis if diagnosed early. Overall, 45.2% of patients were asymptomatic and detected by incidental imaging findings, aligning with published literature that suggests silent progression of disease due to poor diagnosis [1].

This study also observed that 93.2% of participants did not have metastatic RCC at diagnosis, and only 6.8% showed single or multiple metastatic tumors, suggesting an improvement in early diagnosis. However, only 12% of the five-year survival rate is reported in metastatic tumors with multiple organ systems involved. A total of 45.2% of cases showed the presence of tumor necrosis, which makes the prognosis of RCC worse, indicating a more aggressive disease. Patients with tumors less than stage four with dirty necrosis exhibit a short survival rate as compared to ones with ghost necrosis or without necrosis. In ghost tumor necrosis, the number of foci and area of necrosis determines the tumor size and survival rate [35].

Laparoscopic radical surgery accounted for 52.1% of treatment strategies, which indicates the presence of advanced surgical and medical facilities in the region, which improves patient recovery outcomes, particularly in early-stage disease. However, several prognostic biomarkers and histopathological factors impact the prognosis of localized RCC post-surgery [36]. Moreover, the survival outcome of patients using these techniques in the Qassim region still needs to be studied. A five-year recurrence rate from 7.2-61.6% has been reported in the case of ccRCC [37].

The main focus of this study is on the Qassim region, Saudi Arabia, which lags behind in research on renal cancer despite an increasing annual incidence. The findings on clinical and demographic characteristics of RCC in Qassim prerequisites the region-specific studies and targeted public health therapies. 

Limitations of this study include the retrospective design and the single-center setting, which may restrict the generalizability of the findings. Additionally, the reliance on existing medical records for data collection may introduce potential biases. Future research should adopt prospective, multicenter designs to validate these findings. Longitudinal studies examining treatment outcomes and lifestyle interventions are warranted. Furthermore, exploring molecular biomarkers and genetic predispositions may yield deeper insights into the etiology of renal cell carcinoma (RCC). Implementation of regional health education programs focusing on obesity, hypertension, and smoking cessation could serve to mitigate risk factors associated with RCC.

## Conclusions

Extensive information on the global trends and regional distinctions of RCC in the Qassim region has been provided in this study. ccRCC has the highest prevalence among all types of renal cancer, with incidental diagnosis in most cases. Comorbidities such as hypertension and obesity are commonly associated with RCC. RCC management is a complex phenomenon in the region, and further studies are required to investigate longitudinal outcomes, focusing on the long-term effectiveness of medical and surgical therapies and lifestyle interventions.
